# Development of Mn^2+^-Specific Biosensor Using G-Quadruplex-Based DNA

**DOI:** 10.3390/ijms241411556

**Published:** 2023-07-17

**Authors:** Masataka Mizunuma, Mirai Suzuki, Tamaki Kobayashi, Yuki Hara, Atsushi Kaneko, Kazuhiro Furukawa, Yoshiro Chuman

**Affiliations:** Department of Chemistry, Faculty of Science, Niigata University, Niigata 950-2181, Japan; f21j007a@mail.cc.niigata-u.ac.jp (M.M.); f22a024f@mail.cc.niigata-u.ac.jp (M.S.); f23a032a@mail.cc.niigata-u.ac.jp (T.K.); f23a038a@mail.cc.niigata-u.ac.jp (Y.H.); akanekodaigakuyou@gmail.com (A.K.); furukawa@chem.sc.niigata-u.ac.jp (K.F.)

**Keywords:** manganese ion, DNA aptamer, G-quadruplex, IRDAptamer

## Abstract

Metal ions are used in various situations in living organisms and as a part of functional materials. Since the excessive intake of metal ions can cause health hazards and environmental pollution, the development of new molecules that can monitor metal ion concentrations with high sensitivity and selectivity is strongly desired. DNA can form various structures, and these structures and their properties have been used in a wide range of fields, including materials, sensors, and drugs. Guanine-rich sequences respond to metal ions and form G-quadruplex structures and G-wires, which are the self-assembling macromolecules of G-quadruplex structures. Therefore, guanine-rich DNA can be applied to a metal ion-detection sensor and functional materials. In this study, the IRDAptamer library originally designed based on G-quadruplex structures was used to screen for Mn^2+^, which is known to induce neurodegenerative diseases. Circular dichroism and fluorescence analysis using Thioflavin T showed that the identified IRDAptamer sequence designated MnG4C1 forms a non-canonical G-quadruplex structure in response to low concentrations of Mn^2+^. A serum resistance and thermostability analysis revealed that MnG4C1 acquired stability in a Mn^2+^-dependent manner. A Förster resonance energy transfer (FRET) system using fluorescent molecules attached to the termini of MnG4C1 showed that FRET was effectively induced based on Mn^2+^-dependent conformational changes, and the limit of detection (LOD) was 0.76 µM for Mn^2+^. These results suggested that MnG4C1 can be used as a novel DNA-based Mn^2+^-detecting molecule.

## 1. Introduction

The biological macromolecule DNA, which is based on a nucleotide backbone, expresses its function as genes by forming various structures in vivo. DNA forms complementary base pairs based on Watson–Crick-type base pairing. As this complementarity is extremely specific and precise, its development into functional materials has been explored for over a decade [1,2,3,4,5]. In addition, DNA forms duplexes and various higher-order structures [6]. Among them, the G-wire structure, which is formed by the multimerization of guanine-rich DNA sequences in the presence of certain cations, is a higher-order structure formed by DNA chain linkages and has high stability [7,8]. Therefore, the development of functional materials based on G-wires has been pursued, and their application to functional tools, including molecular devices and nanomachines, is expected for future development [9].

Recently, nucleic acid aptamers using DNA and RNA have attracted attention as alternative target recognition molecules to antibodies. RNA/DNA aptamers consisting of single-stranded nucleic acids can specifically bind to their targets and have low antigenicity. Therefore, RNA/DNA aptamers have been extensively studied and applied [10,11]. Compared with antibodies, RNA/DNA aptamers have several advantages, including ease of modification, cost-effectiveness, and short production time. In addition, RNA/DNA aptamers can be generated against various targets, including organic dyes, drugs, amino acids, base analogues, peptides, and numerous proteins. Moreover, the development of DNA-based metal ion detection molecules has been widely explored recently [12].

In cells, endogenous DNA interacts with a limited number of metal ions, such as Na^+^, K^+^, and Mg^2+^. Alternatively, artificially synthesized DNA is expected to be used as a metal ion-responsive molecule because of the following properties [12,13,14]. First, DNA has a negatively charged phosphate backbone that allows electrostatic interactions with metal ions and can be shaped to match specific metal coordination preferences through tertiary DNA folding. Second, DNA is highly stable and can be renatured without losing its affinity to metal ions after denaturation. Third, DNA can be chemically synthesized at low cost, and chemical modifications are possible at arbitrary sites. Fourth, DNA sensors can be designed largely based on DNA secondary structures with minimal knowledge of their 3D folding. Finally, it is possible to selectively isolate and amplify sequences that bind to metal ions via in vitro selection.

Guanine-rich RNA/DNA aptamers can form G-quartets based on Hoogsteen base pairing between four guanine bases in the presence of monovalent cations, such as Na^+^, K^+^, and NH_4_^+^ [15,16,17]. In this case, the cations within the G-quartet stabilize the structure by coordinating with O6 on the guanine bases. G-quadruplex structures are stacked nucleic acid structures found in various organisms, and approximately 700,000 G-quadruplex-forming sites have been found in the human genome [18]. These G-quadruplex structures are primarily found in telomere and promoter regions, and the formation of G-quadruplex structures in the presence of metal ions renders exonucleases resistant to degradation [19,20]. In addition, G-quadruplex DNA acquires high thermal stability. Therefore, the development of functional molecules based on G-quadruplex structures will imbue them with high stability.

Mn^2+^, one of the essential trace elements, functions as a cofactor for enzymes such as kinases, transferases, and decarboxylases in vivo [21]. In addition, Mn^2+^ is involved in various critical biological processes, such as maintaining redox balance in mitochondria, oxygen evolution in chloroplasts, and maintaining cellular homeostasis [22,23,24]. In contrast, chronic exposure to an environment contaminated with Mn^2+^ is involved in Parkinson’s and Alzheimer’s diseases [25,26,27]. These findings highlight the importance of developing methods to detect Mn^2+^ in vivo and in the environment to measure its effects on the environment and human health.

Thus far, the standard methods for metal analysis have been based on high-precision instruments, such as atomic absorption/emission spectrometry, spectrophotometry, and inductively coupled plasma mass spectrometry [12,28,29,30]. Although these techniques are highly sensitive and accurate as industrial standards, they have numerous problems, including high cost, availability only in large laboratories, complex sample preparation, the need for well-trained personnel, and an inability to perform real-time or in situ measurements in the field [31,32]. Therefore, developing easy-to-use and inexpensive methods for the detection of Mn^2+^ is crucial. Although fluorescence-based detection is convenient, the development of selective fluorescent sensors for Mn^2+^ has been limited owing to the paramagnetic properties of Mn^2+^, which quenches fluorescence. A well-known Mn^2+^ sensor using fluorescence emission is a fluorescent molecule based on the ion substitution reaction performed by Canary et al. [33]. Furthermore, the Liang Mn series has been reported as more selective and direct Mn^2+^ molecules [34]. In addition, biopolymer-based Mn^2+^-detection has been developed using different platforms, such as surface plasmon resonance, optical sensors, and graphene nanosheets [35,36,37,38]. However, these molecules have issues concerning their responsiveness to other trace elements and the expense of their complex synthesis.

Previously, we designed an ion-responsive DNA aptamer (IRDAptamer) with guanine bases in the scaffold sequence and randomized sequences in the franking loop region [39]. Simultaneously, we reported an IRDAptamer named M1D-Q5F, which specifically binds to the oncogenic protein phosphatase PPM1D and regulates its activity. Notably, M1D-Q5F forms a propeller-type G-quadruplex structure in the presence of Na^+^ and K^+^, whereas the intensity of the molar ellipticity is different in the presence of the respective metal ions, indicating a different inhibitory activity against PPM1D. These results suggested that the specific cation recognition molecules can be developed using the IRDAptamer library, which has different sequences in their individual loops. In this study, DNAs responsive to Mn^2+^ were screened and identified with SELEX using the IRDAptamer library.

## 2. Results

### 2.1. Evaluation of IRDAptamer Library Responsiveness to Mn^2+^ and Screening of Mn^2+^-Specific IRDAptamers

**Figure 1:**
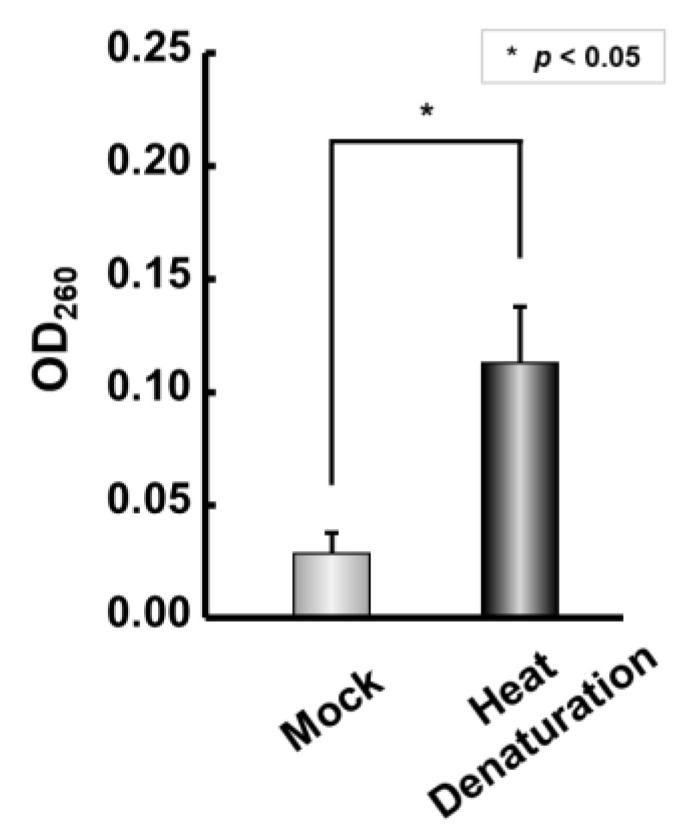
Reconstruction of IRDAptamer library and responsivity for Mn^2+^: Responsivity of IRDAptamer library to 10 mM Mn^2+^. OD_260_ of supernatant including DNA eluted from self-assembled precipitates after heat denaturation was measured. Data represent the mean ± S.D. (n = 3) * (asterisk) indicates *p* < 0.05.

First, the IRDAptamer library used in the screening for the oncogenic protein phosphatase PPM1D was reconstructed for divalent metal ion detection [39]. As previous studies have reported that G-rich sequences self-assemble to form G-wire structures in the presence of certain divalent cations, we evaluated the Mn^2+^ responsiveness of the reconstructed IRDAptamer library [7,9]. After incubation of the IRDAptamer library with 10 mM Mn^2+^, self-assembled precipitates were formed. After washing the self-assembled precipitate with 10 mM Mn^2+^, DNA was eluted via heat denaturation in sterile water, and the DNA in the supernatant was quantitatively evaluated. The absorbance of the supernatant was measured at OD_260_, and it was confirmed that DNA was predominantly eluted from the heat-denatured sample compared to the mock sample, which was not subjected to heat denaturation (Figure 1). These results indicated that the reconstructed IRDAptamer library is responsive to Mn^2+^ and that DNA is eluted from self-assembled IRDAptamer precipitates formed in the presence of Mn^2+^ via heat denaturation. Based on these results, we performed a Mn^2+^-targeted screening using DNA elution via heat denaturation.

**Figure 2:**
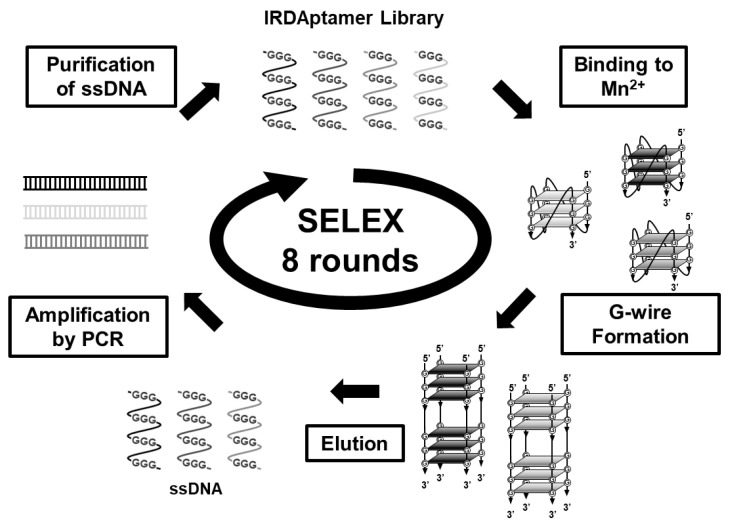
Scheme of screening of Mn^2+^ responsive DNA aptamers from the reconstructed ion-responsive DNA aptamer (IRDAptamer) library.

Next, to identify IRDAptamers that specifically recognize Mn^2+^, we screened IRDAptamers that respond to Mn^2+^ using the in vitro selection (SELEX) method (Figure 2). First, IRDAptamer libraries that responded to Mn^2+^ were incubated with 10 mM Mn^2+^ to form self-assembled precipitates, and DNA was eluted from precipitates via heat denaturation. After 8 rounds of SELEX, the MnG4C1 sequence was identified as the most frequently detected Mn^2+^-recognizing IRDAptamer using a sequence analysis of 31 clones (Table 1 and Table S1).

### 2.2. Evaluation of G-Quadruplex Formation Ability of Mn^2+^-Specific IRDAptamer MnG4C1 Using Thioflavin T

**Figure 3:**
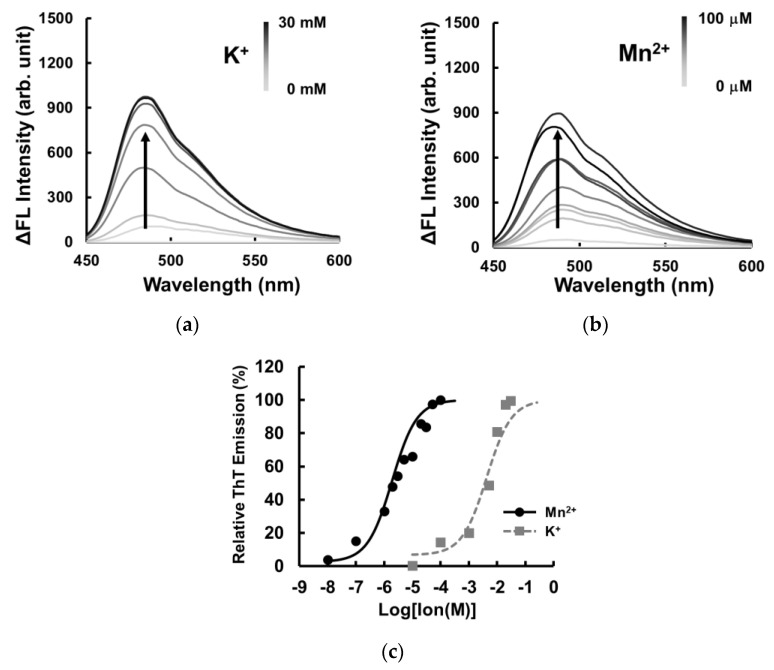
Fluorescent spectrum analysis using Thioflavin T in presence of cation: (**a**) The lines represent the spectrum at concentrations of 0, 0.1, 1, 5, 10, 20, 25, and 30 mM K^+^. (**b**) The lines represent the spectrum at concentrations of 0, 0.1, 1, 2, 3, 5, 10, 20, 30, 50, and 100 µM Mn^2+^. (**c**) Fluorescence intensity of ThT at 492 nm in the presence of each cation (circle, Mn^2+^; square, K^+^).

The MnG4C1 sequence, identified as a Mn^2+^-recognizing IRDAptamer by SELEX, was analyzed for cation responsiveness using Thioflavin T (ThT), a G-quadruplex structure-recognizing fluorescent molecule with a strong emission peak at around 492 nm [40,41]. To characterize the identified aptamer, we evaluated the ability of MnG4C1 to form a G-quadruplex structure using ThT in the cation solution. Fluorescence emission for the identified MnG4C1 in the presence of K^+^, which forms a rigid G-quadruplex structure, revealed that ThT emission at 492 nm increased with K^+^ in a dose-dependent manner (Figure 3a). In addition, ThT fluorescence emission was observed at low concentrations of Mn^2+^ (Figure 3b). However, the half-maximal effective concentration (EC_50_) values calculated using the positive peak of fluorescence emission at 492 nm against each cation were 4.66 ± 1.21 mM for K^+^ and 2.60 ± 0.98 µM for Mn^2+^ (Figure 3c). These data indicated that MnG4C1 formed a G-quadruplex structure in response to Mn^2+^ with a higher sensitivity than K^+^.

### 2.3. Topological Analysis of the Non-Canonical G-Quadruplex Structure of MnG4C1 in Response to Mn^2+^

**Figure 4:**
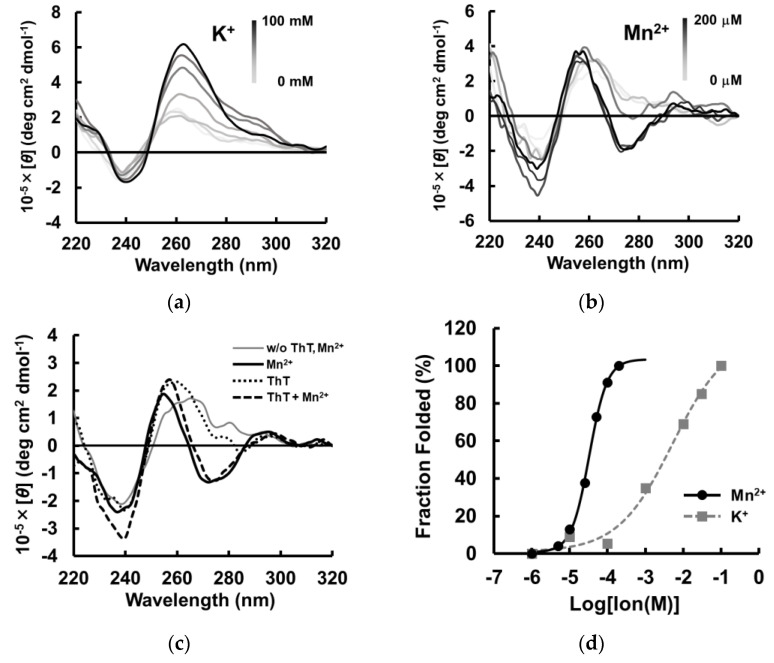
Ion responsiveness of MnG4C1 by CD spectroscopy. (**a**) The lines represent the spectrum at concentrations of 0, 0.01, 0.1, 1, 10, 30, and 100 mM K^+^. (**b**) The lines represent the spectrum at concentrations of 0, 5, 10, 25, 50, 100, and 200 µM Mn^2+^. (**c**) CD spectra in the absence and presence of 3 µM ThT and 100 µM Mn^2+^. (**d**) Normalized CD intensity at 264 nm for K^+^ and at 274 nm for Mn^2+^ (circle, Mn^2+^; square, K^+^).

To evaluate the structural topology of MnG4C1 in the presence of cations, we investigated a conformational analysis using a circular dichroism (CD) spectrometer, which is normally used to characterize the G-quadruplex structure. The G-quadruplex structure exhibits a characteristic CD spectrum depending on its topology [42,43,44]. In the presence of K^+^, CD spectra of MnG4C1 showed a positive peak at 264 nm and a negative peak at 240 nm, indicating that MnG4C1 forms a propeller-type G-quadruplex structure in a K^+^-dependent manner (Figure 4a). In contrast, in the presence of low concentrations of Mn^2+^, a characteristic spectrum with negative peaks at 274 and 240 nm and a positive peak at 260 nm was observed, which was completely different from the spectrum of K^+^ (Figure 4b). For the ThT fluorescence analysis, MnG4C1 showed high emissions because of G-quadruplex formation in a Mn^2+^-dependent manner (Figure 3b); however, ThT functions as an inducer of the G-quadruplex structure, suggesting that the increase in fluorescence intensity in Figure 3b may be because of this. Therefore, we evaluated the CD in the presence of ThT alone and for the ThT and Mn^2+^ condition. The CD spectra of ThT alone showed a spectrum similar to that in the absence of Mn^2+^ and ThT, whereas the spectrum of the ThT and Mn^2+^ condition showed a negative peak at approximately 274 nm in the presence of Mn^2+^ (Figure 4c). These results strongly suggested that the unique structure with a negative peak at 274 nm was not derived from ThT and was formed by the addition of Mn^2+^. Furthermore, the Mn^2+^-dependent increase in the fluorescence intensity in the presence of ThT suggested that the structure formed in the presence of Mn^2+^ is a G-quadruplex structure but not a canonical G-quadruplex structure. EC_50_ values were calculated using the peak values of the structures formed by MnG4C1 in the presence of each cation, and the sensitivity was compared. The EC_50_ value calculated using the positive peak at 264 nm in the presence of K^+^ was 5.25 ± 3.99 mM, and in the presence of Mn^2+^ using the negative peak at 274 nm, it was 32.7 ± 4.5 µM (Figure 4d). These results indicated that MnG4C1 is more sensitive to Mn^2+^ than to K^+^ to form non-canonical G-quadruplex structures, similar to the evaluation of the ability to form G-quadruplex structures using ThT.

### 2.4. Effects of the Loop Region on Mn^2+^ Recognition and Evaluation of Metal Ion Specificity of MnG4C1

**Figure 5:**
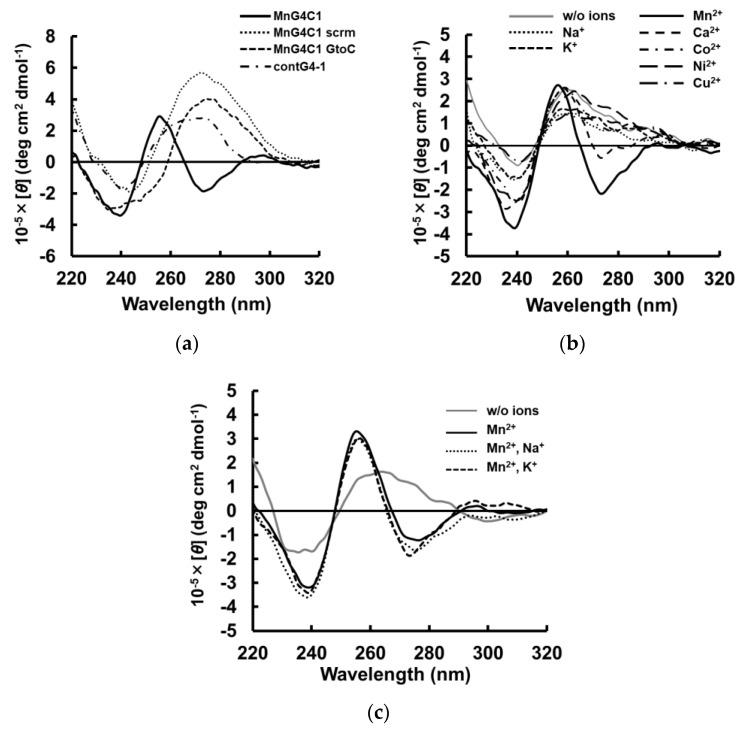
Sequence dependency and cation specificity of MnG4C1 sequence: (**a**) Sequence dependency using MnG4C1 and control sequences in the presence of 100 µM Mn^2+^ by CD spectroscopy; (**b**) Cation specificity of MnG4C1 in the presence of 100 µM each cation as described in figure; (**c**) CD spectroscopy in the presence of 100 µM cations as described.

Next, we evaluated the sequence dependence of MnG4C1 for the recognition of Mn^2+^. First, we designed MnG4C1 GtoC, in which the guanine base in the MnG4C1 scaffold sequence was replaced by a cytosine base, and MnG4C1 scrm, in which the sequence of MnG4C1 was scrambled, and performed CD spectral measurements in the presence of 100 µM Mn^2+^. Both MnG4C1 GtoC and MnG4C1 scrm showed no characteristic negative peaks at 274 nm; however, a positive peak at 280 nm, which is generally exhibited by unstructured single-stranded DNA, was observed (Figure 5a). In addition, contG4-1, which was randomly selected from the IRDAptamer library and has the same G-rich scaffold as MnG4C1, differing only in the sequence of the loop regions, was also measured under the same conditions. The characteristic negative peak at approximately 274 nm was not observed in contG4-1 (Figure 5a). These results indicated that the spectrum with a characteristic negative peak at approximately 274 nm in the presence of 100 µM Mn^2+^ is a MnG4C1 sequence-specific spectrum.

Next, the metal ion specificity of MnG4C1 was evaluated using CD spectrum analysis in the presence of various cations, such as Mg^2+^, Al^3+^, Ca^2+^, Fe^2+^, Co^2+^, Ni^2+^, Cu^2+^, Zn^2+^ and Cd^2+^, in addition to monovalent cations, such as Na^+^ and K^+^, which are known to form the rigid G-quadruplex structure. The CD spectrum in the presence of 100 µM of each cation showed that a spectrum with a characteristic negative peak at 274 nm was observed only in the presence of Mn^2+^, whereas no characteristic negative peak at 274 nm was observed in the presence of other divalent and monovalent cations (Figure 5b). Furthermore, the CD spectrums for several divalent metal ions including Mn^2+^, were analyzed at each maximum residual limitation (MRL) and 10-fold higher MRL concentrations. As a result, no metal ion-dependent changes were observed in the respective spectra at the MRL concentration of each metal ion. On the other hand, at 10-fold MRL concentrations, a minimum peak at 274 nm was observed only for Mn^2+^, while no such characteristic signal change was observed for the other metal ions (Figure S1a–e). These results indicated that MnG4C1 can specifically detect Mn^2+^ at a homogeneous concentration of 100 µM and under a 10-fold MRL concentration of each metal ion. These results showed that the non-canonical G-quadruplex structure formed by MnG4C1 with a negative peak at approximately 274 nm in CD spectrum was induced only in response to Mn^2+^.

In addition, the Mn^2+^ selectivity of MnG4C1 was analyzed via CD spectra in the mixed solution of Mn^2+^ with different metal ions. CD spectra of MnG4C1 in the presence of Mn^2+^ with Na^+^ and K^+^ showed a characteristic negative peak at 274 nm, which was similar to the spectrum in the presence of Mn^2+^ alone (Figure 5c). These results indicated that MnG4C1 can selectively detect Mn^2+^ via the formation of Mn^2+^-specific structures, even in solution with other metal ions, and MnG4C1 may be applicable as a highly sensitive sensor for Mn^2+^. Next, we analyzed the CD spectral changes caused by the addition of Mn^2+^ in the presence of Na^+^ and K^+^ concentrations similar to those in vivo, respectively. As a result, a slight negative peak around 274 nm was observed under the 75 mM Na^+^ condition, however, no characteristic structural changes were observed with or without the presence of 100 µM Mn^2+^ under the conditions of 150 mM extracellular Na^+^ and 150 mM intracellular K^+^ (Figure S2a–c). These data suggested that the guanine-rich tandem sequence in the scaffold structure of IRDAptamer was easily bound by high concentrations of K^+^ and Na^+^ and that the structural changes of MnG4C1 by Mn^2+^ were offset by structural changes by high concentrations of K^+^ or Na^+^.

### 2.5. Structural Stability of the Non-Canonical G-Quadruplex Structure MnG4C1

**Figure 6:**
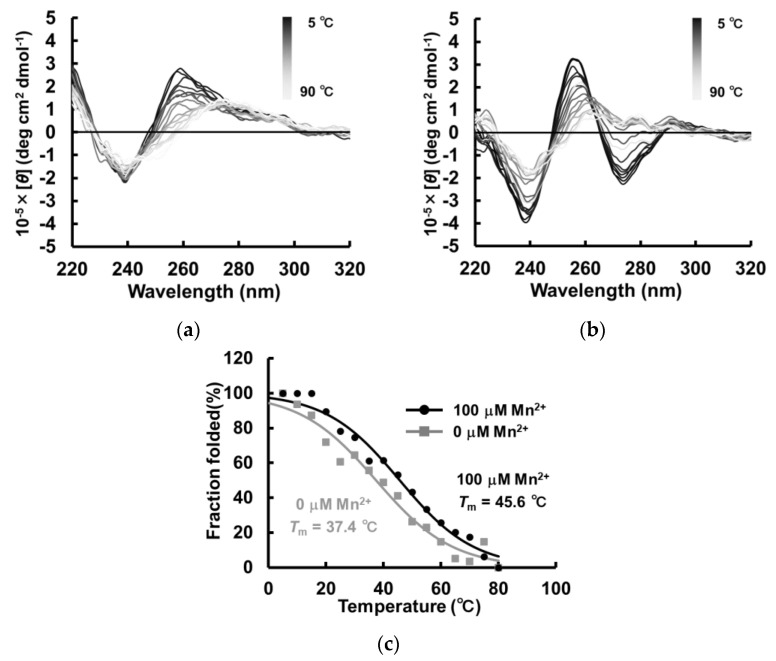
Thermostability analysis of MnG4C1 by CD spectroscopy with measurements taken every 5 °C from 5 °C to 90 °C. (**a**) CD spectra w/o cation condition. (**b**) CD spectra in the presence of 100 µM Mn^2+^. (**c**) Monitoring of heat sensitivity by CD spectra at 260 nm for w/o cation condition and at 274 nm for Mn^2+^ (square, w/o cation; circle, 100 µM Mn^2+^).

Given that the G-quadruplex structure has high thermal stability, we first investigated the thermal sensitivity of MnG4C1 using CD spectroscopy. *T*_m_ values were calculated via temperature-dependent measurements of CD spectra in the absence and presence of Mn^2+^, and the effect of Mn^2+^ on thermal stability was evaluated (Figure 6a,b). Consequently, the *T*_m_ value was calculated to be 37.4 °C in the absence of the cation, whereas the *T*_m_ increased to 45.6 °C in the presence of 100 µM Mn^2+^ (Figure 6c). These results indicated that the thermal stability of MnG4C1 increases in response to low concentrations of Mn^2+^.

**Figure 7:**
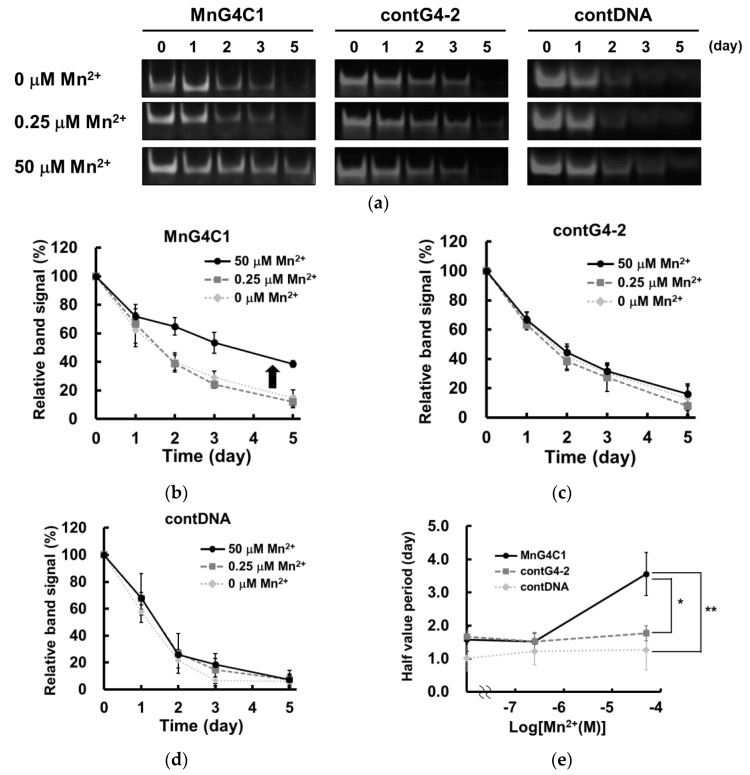
Serum stability analysis of MnG4C1. (**a**) Stability of MnG4C1 and control DNAs in DMEM with 10% FBS at 37 °C for indicated days. (**b**–**d**) Quantitation of the data in (**a**). Data show relative band signal to 0 days and represent mean ± S.D. (n = 3) (rhomb, 0 µM Mn^2+^; square, 0.25 µM Mn^2+^; circle, 50 µM Mn^2+^). (**e**) Half value period of each aptamer in the absence and presence of Mn^2+^ in serum. Data represent the mean ± S.D. (n = 3) (circle, MnG4C1; square, contG4-2; rhomb, contDNA) * and ** indicate *p* < 0.05, and *p* < 0.01, respectively.

In general, the G-quadruplex structure also shows high resistance to nuclease degradation [42,45]. To evaluate this property, we investigated the stability of MnG4C1 in serum. Nuclease resistance assays of MnG4C1 were performed in DMEM containing 10% FBS. In addition, contG4-2 and contDNA, which were MnG4C1 mutants of the loop region and linear short DNA, respectively, were also used as negative controls (Figure 7a). In the nuclease resistance assay, MnG4C1 was predominantly resistant to degradation under 50 µM Mn^2+^ conditions, where it was confirmed to exhibit a characteristic negative peak at 274 nm by CD spectral analysis. However, it was not resistant to degradation in the absence of or under 0.25 µM Mn^2+^ conditions, where MnG4C1 cannot form a G-quadruplex structure (Figure 7a,b). In contrast, contG4-2 and contDNA were degraded with or without Mn^2+^ (Figure 7a,c,d). Based on these results, the half-lives of individual DNA including MnG4C1 indicated that MnG4C1 acquired a relatively long half-life of 3.5 d under 50 µM Mn^2+^, whereas no Mn^2+^-dependent increase in half-life was observed for the other DNAs (Figure 7e). These data indicated that MnG4C1 acquired serum resistance owing to its non-canonical G-quadruplex structure in the presence of Mn^2+^, as contG4-2 and contDNA did not acquire degradation resistance in response to Mn^2+^. Both the thermostability analysis and serum resistance assay results indicated that the non-canonical G-quadruplex structure of MnG4C1 increased in structural stability in response to Mn^2+^, similar to the canonical G-quadruplex structural response to monovalent cations such as K^+^.

### 2.6. FRET Analysis Based on the Structural Transition of MnG4C1

**Figure 8:**
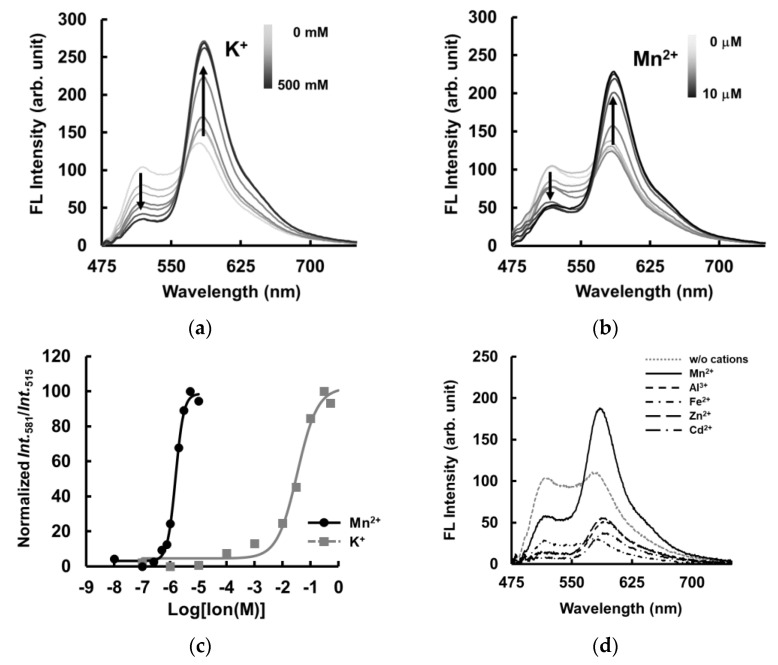

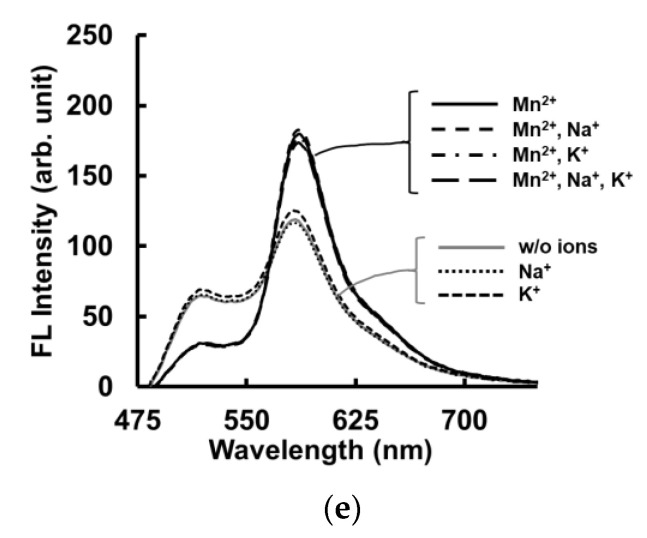
Fluorescent spectrum analysis using MnG4C1 with FAM and TAMRA dyes at its 5′ and 3′ termini for FRET system. (**a**) The lines represent the spectrum at concentrations of 0, 0.1, 1, 10, 30, 100, and 500 mM K^+^. (**b**) The lines represent the spectrum at concentrations of 0, 0.1, 0.25, 0.5, 0.75, 1, 2, 3, 5, and 10 µM Mn^2+^. (**c**) Fluorescence intensity ratio of MnG4C1 using plot of normalized Int.581/Int.515 against K^+^ and Mn^2+^ (circle, Mn^2+^; square, K^+^). (**d**) Fluorescent analysis in the presence of cation concentration of MRL in environmental water (Mn^2+^, 9.1 µM; Al^3+^, 74 mM; Fe^2+^, 36 µM; Zn^2+^, 46 µM; Cd^2+^, 27 nM). (**e**) Fluorescent spectra in the presence of 10 µM multiple cations.

Finally, we aimed to establish a Mn^2+^ detection system based on the conformational change of MnG4C1 to a non-canonical G-quadruplex structure in response to Mn^2+^. To monitor the characteristic conformational change of MnG4C1 from a flexible structure to a rigid non-canonical G-quadruplex structure, we designed a FRET-based Mn^2+^ detection molecule MnG4C1-FT by modifying MnG4C1 with the fluorescent molecules known as FAM and TAMRA dyes at its 5′ and 3′ termini, respectively. FRET analysis of MnG4C1-FT in the presence of K^+^, which has been shown to form a propeller-type G-quadruplex structure using CD analysis, showed that the fluorescence intensity of FAM at 515 nm decreased and that of TAMRA at 581 nm increased in a K^+^ concentration-dependent manner, indicating that FRET was induced (Figure 8a). In contrast, FRET was efficiently induced in low concentrations of Mn^2+^ of up to 10 µM (Figure 8b). These observations suggested that both the 5′ and 3′ ends of MnG4C1 are brought closer together in a Mn^2+^-dependent manner, similar to the K^+^-dependent formation of the propeller-type G-quadruplex structure, forming a more compact structure than in the absence of the cation. To evaluate the sensitivity of FRET efficiency to cations, EC_50_ values were calculated using the TAMRA/FAM values, an indicator of FRET efficiency. The EC_50_ value for K^+^ was 34.1 ± 12.7 mM, whereas for Mn^2+^ it was 1.52 ± 0.17 µM, indicating that MnG4C1 is approximately 20,000-fold more sensitive to Mn^2+^ than to K^+^ in the FRET system (Figure 8c). In addition, the cation specificity of FRET measurements was assessed under MRL conditions, such as the presence of Al^3+^, Fe^2+^, Zn^2+^, Cd^2+^, and Mn^2+^. Under the Al^3+^, Fe^2+^, Zn^2+^ and Cd^2+^ conditions, the fluorescent intensity of FAM and TAMRA was decreased and FRET was not observed; however, FRET was effectively induced in the presence of MRL concentrations of Mn^2+^ (Figure 8d). These data suggested that MnG4C1 can detect Mn^2+^-sensitive FRET signals in the vicinity of the MRL concentration of each metal ion.

Fluorescence measurements of MnG4C1-FT were also performed in the presence of other metal ions, such as Na^+^ and K^+^. In the presence of 10 µM Na^+^ and K^+^, the FRET of MnG4C1-FT was not induced, whereas FRET was predominantly observed in the Mn^2+^-containing condition (Figure 8e, Table S2). The CD spectra of MnG4C1 also showed that MnG4C1 selectively recognized Mn^2+^, even in the presence of Na^+^ and K^+^, indicating that FRET was induced by Mn^2+^ and not Na^+^ or K^+^ at µM-level concentrations.

In addition, FRET analysis was performed in the presence of 10 µM Mn^2+^ with concentrations of K^+^, Na^+^, Mg^2+^, and Ca^2+^ in the biological environment to investigate whether the sensor can be used as an in vivo Mn^2+^ sensor. The results showed that the presence of 10 µM Mn^2+^ in the presence of 0.33 mM Mg^2+^ in the biological environment caused a slight increase in FRET, while the presence of Na^+^ and K^+^ in the biological environment did not cause an increase in FRET (Figure S3a–c). In the presence of Ca^2+^ in the biological environment, the fluorescence of FAM and TAMRA decreased in the presence or absence of Mn^2+^, and FRET could not be observed (Figure S3d). These results suggested that MnG4C1 can induce Mn^2+^-specific conformational changes at low concentrations of metal ions with high selectivity, but in the presence of K^+^ and Na^+^, which are present at high concentrations in the biological environment, the specific conformational changes are offset by the G-quadruplex conformation induced by K^+^ and Na^+^.

## 3. Discussion

In this study, we reconstructed the G-rich DNA aptamer library, IRDAptamer, and performed in vitro selection (SELEX) against Mn^2+^ using the method by which DNA is eluted via heat denaturation from G-wire precipitation. After SELEX screening, the MnG4C1 sequence was identified as a candidate for the Mn^2+^ sensitive sequence. Fluorescence spectrum analysis using ThT showed that MnG4C1 increased emissions at 492 nm in a Mn^2+^-dependent manner, similar to the K^+^ response that induces the canonical G-quadruplex structure. CD measurements showed that MnG4C1 formed a characteristic negative peak at approximately 274 nm in the presence of a low concentration of Mn^2+^, although it showed a propeller-type G-quadruplex structure in response to K^+^. A degradation analysis in serum suggested that MnG4C1 acquired serum resistance in the presence of Mn^2+^, and no other DNAs were found to be resistant to serum in response to Mn^2+^. In addition, a thermostability analysis using CD showed that MnG4C1 acquired thermostability with the addition of Mn^2+^, indicating the characteristics of the G-quadruplex. Finally, a FRET analysis showed that FRET was induced in response to low concentrations of Mn^2+^. These data suggested that MnG4C1 can be used as a novel DNA-based Mn^2+^ detection molecule (Table 2).

In this study, MnG4C1 was identified from the IRDAptamer library by forming self-assembled precipitates under high Mn^2+^ concentration conditions. Therefore, MnG4C1 may form a self-assembled macromolecule such as G-wire in the presence of a high concentration of Mn^2+^ [7,8,46]. Recently, the development of functional materials using DNA hydrogel has attracted much attention [47]. DNA hydrogels, known as DNA microgels or supramolecular gels, have the dual property of the biological function of DNA and the structural characteristics of a gel. Therefore, numerous functional molecules based on DNA hydrogels have been reported [48,49]. Some research groups reported the detection and removal of molecules of heavy metal ions from the environment as applications of DNA hydrogels [50,51]. These molecules utilize the inherent function of DNA sequences by forming a DNA hydrogel to detect and remove heavy metal ions present in the environment. Thus, a DNA hydrogel using MnG4C1, which responds to Mn^2+^, may be used to detect or capture molecules of Mn^2+^ in the environment.

The topology of G-quadruplex structures, in contrast to Watson–Crick base pairs, form characteristic stacked structures and can be determined by measuring their CD spectra [42,43,44]. The structures with a positive peak at approximately 260 nm and a negative peak at approximately 240 nm are classified as propeller-type G-quadruplexes, and the structure formed by MnG4C1 in response to K^+^ corresponds to this category. However, in addition to these peaks, a characteristic negative peak at approximately 274 nm was observed in the presence of a low concentration of Mn^2+^. The spectrum showing this characteristic negative peak is similar to the CD spectrum of A-type DNA, which is normally found in GC-rich sequences [52,53,54]. Furthermore, a CD spectrum transition was induced by multimerization from the G-quadruplex to the G-wire structure in the presence of specific cations [55,56,57]. In addition, divalent metal ions, including Mn^2+^, destabilize some G-quadruplex structures formed in the presence of Na^+^ and K^+^ as a general property of G-quadruplex structures [17,58,59]. However, in the present study, the fluorescence sensitivity analysis using ThT and the presence of G-quadruplex properties, such as nuclease resistance and thermal stability, suggested that MnG4C1 acquires similar structures and properties to G-quadruplex DNA.

Fluorescence-labeled MnG4C1 induced FRET by the addition of Mn^2+^ and K^+^, which forms the canonical G-quadruplex structure. As the donor and acceptor molecules must be close to the Förster distance of 40 to 60 Å for FRET to be induced, the addition of Mn^2+^ induced a conformational change that brought the termini of MnG4C1 close to each other. The EC_50_ value, using the FRET system as a K^+^ detection tool based on TBA in the previous study, was 0.29 mM and 34 mM for MnG4C1. This showed that the selectivity of MnG4C1 for K^+^ was a reduced response [60,61]. In addition, given that FRET was induced sensitively for low concentrations of Mn^2+^, even in the presence of K^+^ and Na^+^, MnG4C1 can be used as Mn^2+^ detection molecules in the presence of other cations [62,63,64]. DNAzyme was reported to be a DNA-based Mn^2+^ detection molecule by Li et al. In this study, the in vitro selection of DNAzyme using Mn^2+^ alone was performed; however, it still yielded the known motifs, and the specificity was low [65]. Li et al. also reported DNAzyme had relatively high specificity for metal cations, including Mn^2+^, under different pH conditions [66]. In our study, we developed Mn^2+^-specific molecules using the originally designed IRDAptamer library as a differential approach and demonstrated that MnG4C1 induced specific conformational changes in response to Mn^2+^. Collectively, MnG4C1 can be used as a DNA-based Mn^2+^ detection molecule with high sensitivity.

FRET analysis using MnG4C1 showed that FRET was sensitively observed for Mn^2+^ in the µM-order concentration range and that the 5′ end and the 3′ end of MnG4C1 were shown to approach each other and form a compact structure in a Mn^2+^-dependent manner. This suggests that MnG4C1 can be used as a highly sensitive sensor of Mn^2+^. On the other hand, FRET of MnG4C1 was not observed for Mn^2+^ in the presence of Na^+^ and K^+^ in the biological environment, although an enhanced FRET signal that could detect Mn^2+^ was observed in the presence of Mg^2+^ in the biological environment. This may be due to the fact that Na^+^ and K^+^ at high concentrations easily form G-quadruplex structures in conventionally guanine-rich sequences, and the structural changes caused by the addition of Mn^2+^ were offset by the addition of high concentrations of Na^+^ and K^+^. It was suggested that aptamers with higher selectivity for Mn^2+^ could be isolated by screening with depletion of aptamers that bind K^+^ and Na^+^ in the SELEX process using a secondary library based on MnG4C1 since MnG4C1 works as a Mn^2+^-specific sensor at similar concentrations in this study. This screening process using a MnG4C1-derived secondary library may enable the isolation of aptamers with higher selectivity for Mn^2+^ [67,68]. These methods may lead to the development of aptamer sensors that can discriminate Mn^2+^ even in the presence of various ion mixtures in biological environments.

CD spectral and FRET analyses showed that MnG4C1 forms a compact and stable non-canonical G-quadruplex structure in response to Mn^2+^; however, the detailed structure remains elusive. Thus, detailed structural elucidation is required. Crystallography and NMR are commonly used for structural analysis; however, NMR can provide structural analysis in the solution state [69,70,71]. In addition, G-quadruplex structure analysis based on Raman spectroscopy has recently attracted attention [72,73]. The elucidation of the crystallization of MnG4C1 will be useful to clarify the Mn^2+^ recognition mechanism using MnG4C1, and for developing a more sensitive Mn^2+^ detection sensor.

In summary, MnG4C1 showed a high degree of sensitivity for the Mn^2+^ among different metal ions. Therefore, it could be applied as a detection tool for Mn^2+^ present in the environment or matrix. In addition, as we have developed an IRDAptamer targeting Mn^2+^ in this study, it is possible to develop IRDAptamers that sensitively respond to different metal ions. This may lead to the development of detection molecules for essential trace elements for which no useful detection molecules have been developed.

## 4. Materials and Methods

### 4.1. DNA and Sample Preparation

The oligodeoxynucleotides used in this study were obtained from Eurofins Genomics (Tokyo, Japan) after purification to OPC grade or HPLC grade, which is only for fluorescent dye-attached DNA, and used without any further purification. The following DNA sequences were used for all the studies. Reconstructed IRDAptamer library: 5′-AGCGTCGAATACCACTACAC-AGGG-N6-TTAGGG-N6-TTAGGG-N6-TTAGGG-TAATGGAGCTCGTGGTCAGC-3′, forward primer: 5′- AGCGTCGAATACCACTACAC-3′, reverse primer: 5′-GCTGACCACGAGCTCCATTA-3′, biotinylated reverse primer: 5′-biotin-GCTGACCACGAGCTCCATTA-3′, MnG4C1: 5′-AGGG-GGGGAG-TTAGGG-CGCACG-TTAGGG-GTGCTA-TTAGGG-3′, MnG4C1-FT: 5′-FAM-AGGG-GGGGAG-TTAGGG-CGCACG-TTAGGG-GTGCTA-TTAGGG-TAMRA-3′ All other chemical reagents were obtained from Fuji-Film, Wako Pure Chemical Corporation and used without further purification.

### 4.2. Screening of DNA Aptamer Using SELEX Method

The IRDAptamer library was first mixed with 10 mM Mn^2+^ and incubated for 30 min. After the incubation, the sample was centrifugated, and the supernatant was removed from the tube. As a wash buffer, 1 mL of 10 mM Mn^2+^ was added and washed once the precipitation of G-wire occurred specifically in response to Mn^2+^. A total of 50 µL H_2_O was added to the precipitation and incubated on 95 °C heat blocks for 10 min to dissociate DNA and Mn^2+^. After the dissociation from Mn^2+^, eluted IRDAptamer was precipitated with 100% EtOH and washed with 70% EtOH. Purified G-quadruplex DNA aptamer was dissolved in 100 µL of PCR mixture (1× KOD buffer, 1 mM MgSO_4_, 0.2 µM forward primer and biotinylated reverse primer, 0.2 mM dNTPs, and 0.02 U/µL KOD plus ver.2) and amplified by PCR reaction (TOYOBO, Osaka, Japan). The PCR conditions were 3 min at 95 °C and 25 cycles of 30 s at 95 °C, 30 s at 58 °C, 15 s at 68 °C, and a final step of 2 min at 68 °C after the last cycle. Amplificated DNA was monitored by electrophoresis on 4% agarose gel in TBE buffer. A total of 18.5 µL of 4 M NaCl and 5 µL of NeutrAvidin beads were added to 85 µL PCR product to capture the amplified biotinylated PCR product. After 10 min incubation with rotation at room temperature, the sample was centrifugated, and the supernatant was removed. A total of 1 mL H_2_O was added, and the beads that bound the PCR product were washed three times. To separate the sense strand of the dsDNA from the immobilized biotinylated anti-sense strand, 40 µL of 0.1 M NaOH was added to the NeutrAvidin beads and incubated with rotation for 20 min. This mixture was centrifugated, and the supernatant was neutralized with 4 µL of 1 M HCl. The separated sense DNA strand was precipitated with EtOH, washed with 70% EtOH, and dissolved in 100 µL H_2_O. Then, 90 µL of the solution was used as a DNA library for the next round of the selection cycle. The selected DNA pool from the 8th round of SELEX was amplified with forward and non-biotinylated reverse primers, cloned into a pTA2 vector, and transformed into *E. coli* DH5α (TOYOBO, Osaka, Japan). The plasmid DNA was sequenced and identified as MnG4C1.

### 4.3. Fluorescent Measurements for Thioflavin T

Thioflavin T (ThT, 3,6-dimethyl-2-(4-dimethylaminophenyl) benzo-thiazolium cation) was obtained from Fuji-Film, Wako Pure Chemical Corporation and used without further purification. Fluorescent measurements of ThT were performed at a 3 µM concentration of ThT in 1 mM aptamer and 50 mM Tris-HCl (pH 7.5) buffer on a JASCO FP-8300 spectrofluorometer (JASCO, Tokyo, Japan) at room temperature. The fluorescent emissions were collected between 450 nm and 600 nm, and the excitation wavelength of ThT was set at 425 nm. To evaluate the ability to form the G-quadruplex structure, half-maximal effective concentration (EC_50_) values were calculated using fluorescent intensity data at 492 nm and fit with GraphPad Prism8 software according to the two-state Hill equation (Equation (1))

F = F_max_ + (F_min_ − F_max_)/{1 + ([M^+^]/[EC_50_])*^n^*} (1)

where F_max_ is the fluorescent intensity signal corresponding to the fully folded conformation, F_min_ is the fluorescent intensity in non-ionic conditions, [M^+^] is the cation concentration, and *n* is the Hill coefficient.

### 4.4. Circular Dichroism Spectroscopy

Circular dichroism (CD) studies were carried out on a JASCO J-725 spectrometer (JASCO, Tokyo, Japan) attached to a Peltier temperature controller (model; PTC-348WI). The oligonucleotide samples were 5 µM, and all samples were heated at 95 °C for 3 min and cooled down slowly to room temperature using a thermal cycler to form G-quadruplex structures. The spectra were measured in the wavelength range of 220-320 nm using a quartz cuvette with a 1.0 mm path length. The scanning speed was set to 200 nm/min. Each trace was measured at a 0.5 nm data pitch and 1 nm bandwidth and presented as the averages of 5 scans. All of the CD spectra were corrected for signal contribution from the buffer, and the observed ellipticities were converted to mean residue ellipticity. To estimate the affinity between the aptamers and cations, EC_50_ values were calculated using GraphPad Prism8 software. EC_50_ values were calculated using normalized molar ellipticity (*θ*) data at 264 nm for K^+^ and at 274 nm for Mn^2+^ according to the two-state Hill equation (Equation (2))

*θ* = *θ*_*max*_ + (*θ*_*min*_ − *θ_max_*)/{1 + ([M^+^]/[EC_50_])^*n*^} (2)

where *θ*_max_ is the CD signal corresponding to the fully folded MnG4C1 signal, *θ*_min_ indicates the CD signal in the condition without cations, [M^+^] is the cation concentration, and *n* is the Hill coefficient.

The following sequences were used as controls for sequence dependency. MnG4C1 scrm, which was used as a flexible structure: 5′-CGATGTGGTGGGGTGGGAAGCGTTAGTGTAGGCGGCGAAG-3′; MnG4C1 GtoC, which was used as a flexible structure, 5′-ACCCGGGGAGTTACCCCGCACGTTACCCGTGCTATTACCC-3′; and contG4-1 which was used as a canonical G-quadruplex structure, 5′-AGGGATTTGTTTAGGGGGTCTCTTAGGGATAGGGTTAGGG-3′.

### 4.5. Thermostability Analysis Using CD Spectroscopy

Thermostability analyses were carried out on a JASCO J-725 spectrometer, as described above. For the determination of *T*_m_, CD spectra were recorded over a temperature range of 5–90 °C with a step size of 5 °C. Acquired spectra were baseline-corrected for signal contribution due to the buffer, and the observed CD was converted to mean molar ellipticity. *T*_m_ values were calculated at the peak ellipticity of each spectrum using GraphPad Prism8 software.

### 4.6. Serum Stability

To evaluate the serum stability of the identified aptamer, TAMRA-labeled DNAs were incubated in DMEM (Nacalai Tesque, Japan) with 10% FBS for 0, 1, 2, 3, or 5 days in an air incubator at 37 °C. As controls, the following sequences were used in the same conditions: contG4-2, 5′-GAGGTAATTGTTAGGGGCGTTGTTAGGGTGGGACTTAGGG-3′, contDNA, 5′-ATGACCATGACCCTCCACAC-3′. After each time point, samples were treated with a phenol-chloroform solution, which quenched nuclease activity. The serum stability of the DNA was determined by 12% polyacrylamide gel electrophoresis. Gels were analyzed using a BZ-X810 fluorescence microscope (Keyence, Osaka, Japan), and the band signal was quantitively evaluated by the ImageJ method. The half-value period of each aptamer was calculated using GraphPad Prism8 software.

### 4.7. Fluorescent Measurement for FRET

Fluorescent measurements for FRET were also carried out with a JASCO FP-8300 spectrofluorometer and performed with an 0.2 µM oligonucleotide probe in H_2_O at room temperature. The excitation wavelength in the fluorescent measurements was 460 nm for FAM. The efficiency of FRET was expressed using an emission band of FAM at 515 nm and an emission band of TAMRA at 581 nm against the concentrations of K^+^ and Mn^2+^. To determine the FRET efficiency, the normalized fluorescent intensity rate (TAMRA/FAM) at 515 nm for FAM emissions and 581 nm for TAMRA emissions were fit with GraphPad Prism8 software according to the two-state Hill equation (Equation (3))

R = R_max_ + (R_min_ − R_max_)/{1 + ([M^+^]/[EC_50_])*^n^*} (3)

where R_max_ is the fluorescent intensity signal rate corresponding to the fully folded conformation, R_min_ is the fluorescent intensity rate in non-ionic conditions, [M^+^] is the cation concentration and *n* is the Hill coefficient. The limit of detection (LOD) was defined as the lowest signal obtained (bottom value; 0.25 µM Mn^2+^) plus three times its standard deviation (bottom value + 3× SD value). Triplicate measurements were performed for each concentration.

### 4.8. Statistical Analysis

Results are shown as mean ± S.D. Data shown in the study were obtained from independent experiments repeated at least three times. Statistical analyses were performed using an unpaired, two-tailed Student’s *t*-test. Data were considered statistically significant when the value of *p* was <0.05.

## Figures and Tables

**Table 1:** Identified IRDAptamer sequence targeting Mn^2+^ using SELEX method. N indicates randomized sequence of A, T, G or C.

Name	Sequence	Frequency
MnG4C1	5′-AGGG-GGGGAG-TTAGGG-CGCACG-TTAGGG-GTGCTA-TTAGGG-3′	3
Others	5′-AGGG-NNNNNN-TTAGGG-NNNNNN-TTAGGG-NNNNNN-TTAGGG-3′	28
Total		31

**Table 2:** Summary of cation specificity and apparent *K*_D_ values of MnG4C1.

Method	Cations	Apparent *K*_D_ (EC_50_) Value
ThT fluorescent analysis	K^+^	4.66 ± 1.21 mM
	Mn^2+^	2.60 ± 0.98 µM
CD analysis	K^+^	5.25 ± 3.99 mM
	Mn^2+^	32.7 ± 4.5 µM
FRET analysis	K^+^	34.1 ± 12.7 mM
	Mn^2+^	1.52 ± 0.17 µM

## Data Availability

Not applicable.

## Supplementary Materials

The following supporting information can be downloaded at: https://www.mdpi.com/article/10.3390/ijms241411556/s1.
