# Supplementary material for: Development of Mn2+-Specific Biosensor Using G-Quadruplex-Based DNA
Source: Int J Mol Sci. 2023 Jul 17;24(14):11556. doi: 10.3390/ijms241411556 (PMC10380348; doi:10.3390/ijms241411556)
Supplement: Supplementary file 1 [file ijms-24-11556-s001.zip › ijms-2493950-supplementary.pdf]

Table S1. All sequence data analyzed in the 8 rounds of SELEX.

Name	Sequence	Frequency
MnG4C1	5′-AGGG-GGGGAG-TTAGGG-CGCACG-TTAGGG-GTGCTA-TTAGGG-3′	3
	5′-AGGG-CAACGT-TTAGGG-TGCATT-TTAGGG-CGTCGT-TTAGGG-3′	1
	5′-AGGG-CAGGTC-TTAGGG-TCCTCT-TTAGGG-CTTTGT-TTAGGG-3′	1
	5′-AGGG-ACAAGC-TTAGGG-CGTCCA-TTAGGG-TAACAG-TTAGGG-3′	1
	5′-AGGG-CCACTT-TTAGAA-CCAGG-TTAGGG-TAATAC-TTAGGG-3′	1
	5′-AGGG-CTGAGT-TTAGGG-TAGTAG-TTAGGG-TCTAGA-TTAGGG-3′	1
	5′-AGGG-AAGGGA-TTAGGG-GGATCG-TTAGGG-TTCGCG-TTAGGG-3′	1
	5′-AGGT-CCGGCG-TTAGGG-GCTTTG-TTAGGG-GTACTT-TTAGGG-3′	1
	5′-AGGG-GGAGAG-TTAGGG-CTGAGA-TTAGGG-CATGTA-TTAGGG-3′	1
	5′-AGGG-AGGGAG-TTAGGG-GGAGTT-TTAGGG-TCGTGT-TTAGGG-3′	1
	5′-AGGG-AATTAC-TTAGGG-TGGGGG-TTAGGG-CGTTTG-TTAGGG-3′	1
	5′-AGGG-TCTATT-TTAGGG-CCCCCA-TTAGGG-AGGTTG-TTAGGG-3′	1
	5′-AGGG-TCAGGA-TTAGGG-CCGCTA-TTAGGG-TTTCCA-TTAGGG-3′	1
	5′-AGGG-AGCAAG-TTAGGG-AATTCC-TTAGGG-TGCATG-TTAGGG-3′	1
	5′-AGGG-ATTTGT-TTAGGG-GGTCTC-TTAGGG-ATAGGG-TTAGGG-3′	1
	5′-AGGG-GCCCTA-TTAGGG-GTTCCT-TTAGGG-ATCGCG-TTAGGG-3′	1
	5′-AGGG-GCTTAT-TTAGGG-CTAACG-TTAGGG-ATTCTC-TTAGGG-3′	1
	5′-AGGG-TAATCT-TTAGGG-CTGCTC-TTAGGG-AGGTGG-TTAGGG-3′	1
	5′-AGGG-GCGCAC-TTAGGG-TACACG-TTAGGG-CTTTTG-TTAGGG-3′	1
	5′-AGGG-ATATTA-TTAGGG-GCAGGT-TTAGGG-TTGTCT-TTAGGG-3′	1
	5′-AGGG-CAAACG-TTAGGG-CGCCTC-TTAGGG-GGGTAG-TTAGGG-3′	1
	5′-AGGG-ACACCT-TTAGGG-TTGTC-TTAGGG-GTATGA-TTAGGG-3′	1
	5′-AGGG-AGTCTA-TTAGGG-CCCAAT-TTAGGG-ATCTTT-TTAGGG-3′	1
	5′-AGGG-TTTAGT-TTAGGG-ATAGTG-TTAGGG-GGCATC-TTAGGG-3′	1
	5′-AGGG-TGTACC-TTAGGG-TGCGCG-TTAGGG-AACATA-TTAGGG-3′	1
	5′-AGGG-GAGCG-TTAGGG-TGCTTG-TTAGGG-3′	1
	5′-AGGG-GGATCG-TTAGGG-TTTTTG-TTAGGG-3′	1
	5′-AGGG-GTTGGTT-TTAGGG-GTTTAG-TTAGGG-3′	1
	5′-AGGG-ACTGAATTAGT-TTAGGG-3′	1
Total		31

Table S2. FRET efficiency in the presence of 10 μM Na^+, K^+ and Mn^{2+} using MnG4C1-FT.

**Cation species**	**FRET efficiency ($Int._{581}$/$Int._{515}$)**
Mock	1.88
Na^+	1.81
K^+	1.87
Mn^{2+}	5.96
Na^+ + Mn^{2+}	5.98
K^+ + Mn^{2+}	5.98
Na^+ + K^+ + Mn^{2+}	5.76

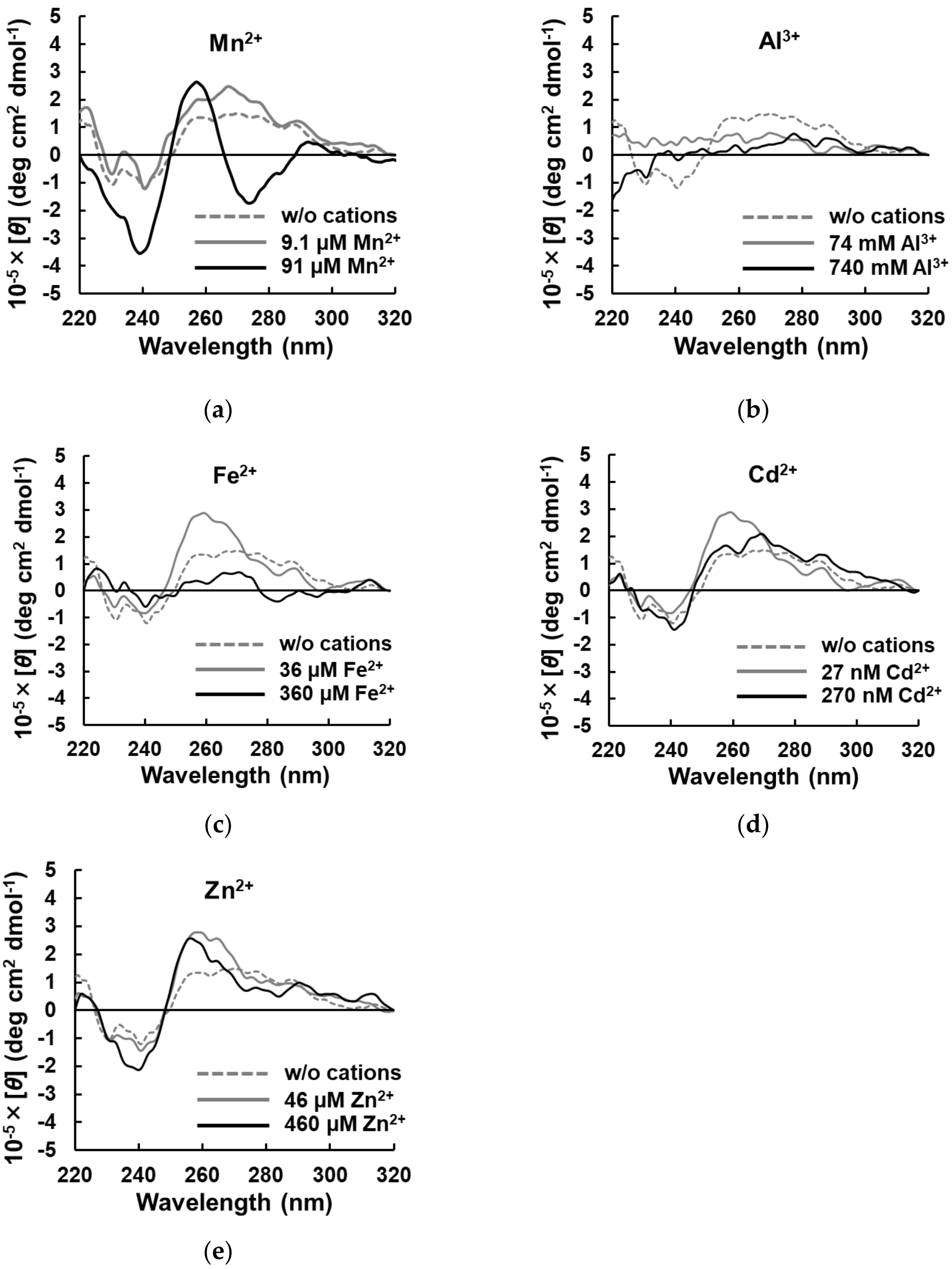

Figure S1. Cation specificity analysis based on the concentration of maximum residual limitation (MRL) and 10-fold MRL using CD spectroscopy. (a) CD spectra in the presence of Mn^{2+} (Mn^{2+}, 9.1 μM for gray and 91 μM black). (b) CD spectra in the presence of Al^{3+} (Al^{3+}, 74 mM for gray and 740 mM for black). (c) CD spectra in the presence of Fe^{2+} (Fe^{2+}, 36 μM for gray and 360 μM for black). (d) CD spectra in the presence of Cd^{2+} (Cd^{2+}, 27 nM for gray and 270 nM for black). (e) CD spectra in the presence of Zn^{2+} (Zn^{2+}, 46 μM for gray and 460 μM for black).

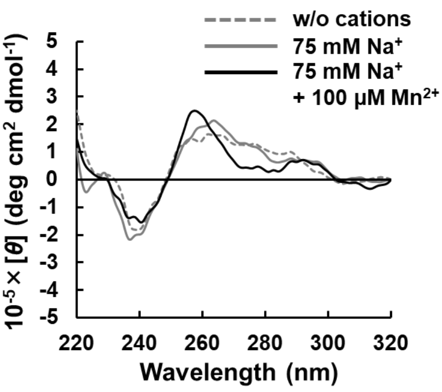

(**a**)

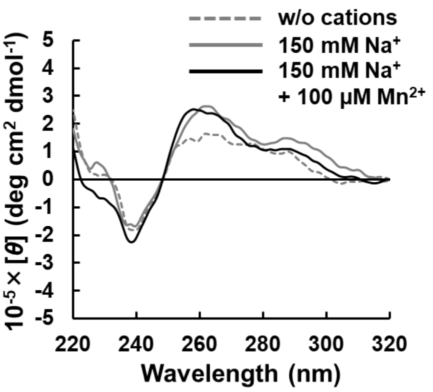

(**b**)

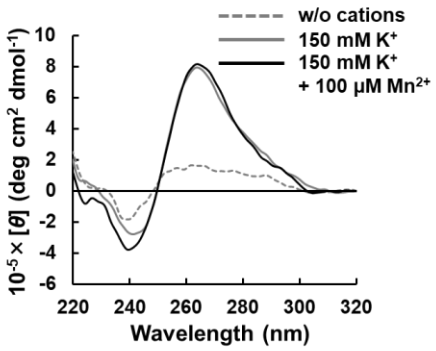

(**c**)

Figure S2. CD spectrum analysis in the presence of Na^+ and K^+. (a) CD spectrum in the presence of 75 mM Na^+ and 100 μM Mn^{2+} (75 mM Na^+, gray; 75 mM Na^++100 μM Mn^{2+}, black). (b) CD spectrum in the presence of 150 mM Na^+ and 100 μM Mn^{2+} (150 mM Na^+, gray; 150 mM Na^++100 μM Mn^{2+}, black). (c) CD spectrum in the presence of 150 mM K^+ and 100 μM Mn^{2+} (150 mM K^+, gray; 150 mM K^++100 μM Mn^{2+}, black).

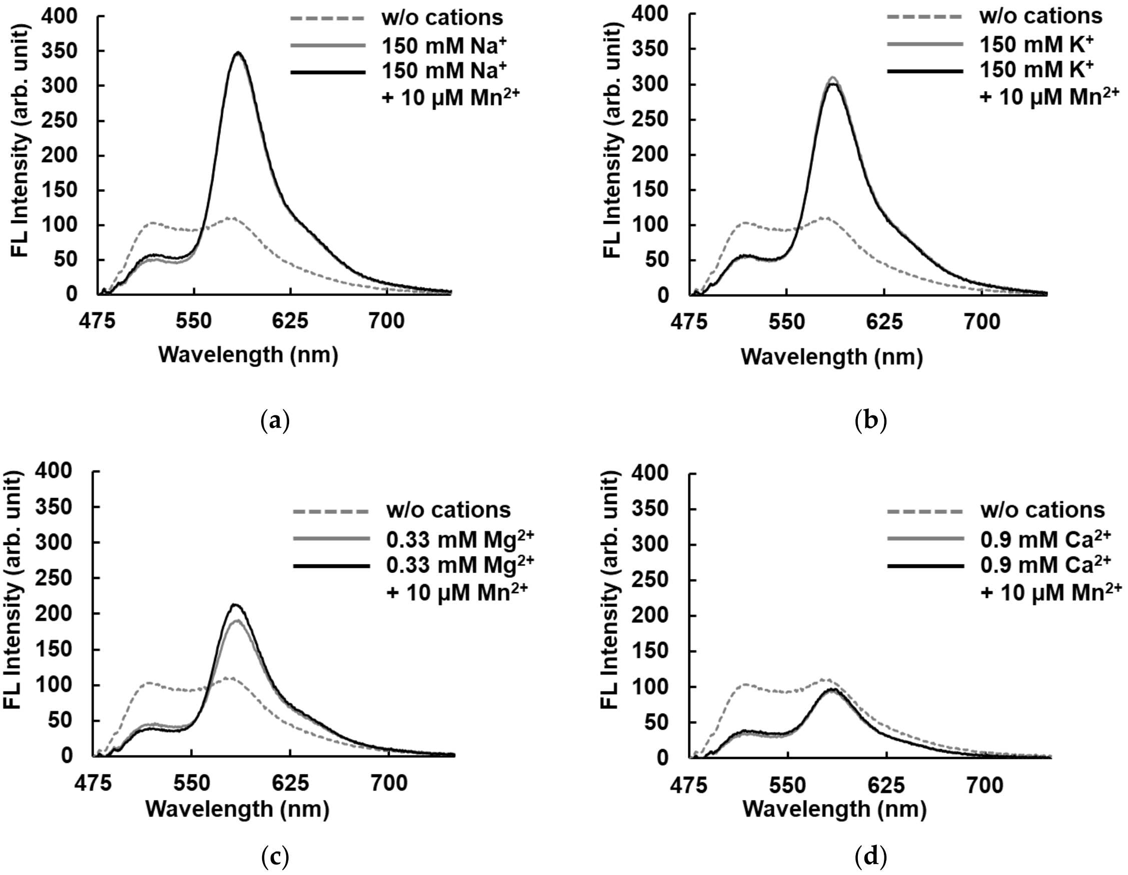

Figure S3. FRET analysis based on the concentration of inter/intra-cellular conditions. (a) Fluorescent spectra in the presence of 150 mM Na^+ and 10 μM Mn^{2+} (150 mM Na^+, gray; 150 mM Na^+ +10 μM Mn^{2+}, black). (b) Fluorescent spectra in the presence of 150 mM K^+ and 10 μM Mn^{2+} (150 mM K^+, gray; 150 mM K^+ +10 μM Mn^{2+}, black). (c) Fluorescent spectra in the presence of 0.33 mM Mg^{2+} and 10 μM Mn^{2+} (0.33 mM Mg^{2+}, gray; 0.33 mM Mg^{2+} +10 μM Mn^{2+}, black). (d) Fluorescent spectra in the presence of 0.9 mM Ca^{2+} and 10 μM Mn^{2+} (0.9 mM Ca^{2+}, gray; 0.9 mM Ca^{2+} +10 μM Mn^{2+}, black).
